# Ibero–American Consensus on Low- and No-Calorie Sweeteners: Safety, Nutritional Aspects and Benefits in Food and Beverages

**DOI:** 10.3390/nu10070818

**Published:** 2018-06-25

**Authors:** Lluis Serra-Majem, António Raposo, Javier Aranceta-Bartrina, Gregorio Varela-Moreiras, Caomhan Logue, Hugo Laviada, Susana Socolovsky, Carmen Pérez-Rodrigo, Jorge Antonio Aldrete-Velasco, Eduardo Meneses Sierra, Rebeca López-García, Adriana Ortiz-Andrellucchi, Carmen Gómez-Candela, Rodrigo Abreu, Erick Alexanderson, Rolando Joel Álvarez-Álvarez, Ana Luisa Álvarez Falcón, Arturo Anadón, France Bellisle, Ina Alejandra Beristain-Navarrete, Raquel Blasco Redondo, Tommaso Bochicchio, José Camolas, Fernando G. Cardini, Márcio Carocho, Maria do Céu Costa, Adam Drewnowski, Samuel Durán, Víctor Faundes, Roxana Fernández-Condori, Pedro P. García-Luna, Juan Carlos Garnica, Marcela González-Gross, Carlo La Vecchia, Rosaura Leis, Ana María López-Sobaler, Miguel Agustín Madero, Ascensión Marcos, Luis Alfonso Mariscal Ramírez, Danika M. Martyn, Lorenza Mistura, Rafael Moreno Rojas, José Manuel Moreno Villares, José Antonio Niño-Cruz, María Beatriz P. P. Oliveira, Nieves Palacios Gil-Antuñano, Lucía Pérez-Castells, Lourdes Ribas-Barba, Rodolfo Rincón Pedrero, Pilar Riobó, Juan Rivera Medina, Catarina Tinoco de Faria, Roxana Valdés-Ramos, Elsa Vasco, Sandra N. Wac, Guillermo Wakida, Carmina Wanden-Berghe, Luis Xóchihua Díaz, Sergio Zúñiga-Guajardo, Vasiliki Pyrogianni, Sérgio Cunha Velho de Sousa

**Affiliations:** 1 Institute of Biomedical and Health Sciences (IUIBS), University of Las Palmas de Gran Canaria (ULPGC), Las Palmas de Gran Canaria 35016, Spain; adriana.ortiz@ulpgc.es (A.O.-A.); aalvfal@gobiernodecanarias.org (A.L.Á.F.); catarinaa711@gmail.com (C.T.d.F.); 2 Spanish Academy of Nutrition and Food Sciences (AEN), Barcelona 08029, Spain; jaranceta@unav.es (J.A.-B.); gvarela@ceu.es (G.V.-M.); carmen.perezr@ehu.eus (C.P.-R.); 3 Nutrition Research Foundation (FIN), University of Barcelona Science Park, Barcelona 08028, Spain; fin@fin.pcb.ub.es; 4 CIBER Fisiopatología de la Obesidad y Nutrición (CIBER OBN), Instituto de Salud Carlos III, Madrid 28029, Spain; marcela.gonzalez.gross@upm.es (M.G.-G.); mariarosaura.leis@usc.es (R.L.); 5 Research Center for Biosciences and Health Technologies-CBIOS, Universidade Lusófona de Humanidades e Tecnologias, Lisboa 1749-024, Portugal; ajasr@hotmail.com; 6 Spanish Society of Community Nutrition (SENC), Barcelona 08029, Spain; 7 Department of Physiology, University of the Basque Country (UPV/EHU), Leioa (Bizkaia) 48940, Spain; 8 Spanish Nutrition Foundation (FEN), Madrid 28010, Spain; 9 Department of Pharmaceutical & Health Sciences, School of Pharmacy, CEU San Pablo University, Boadilla del Monte (Madrid) 28668, Spain; 10 Nutritional Innovation Centre for Food & Health (NICHE), School of Biomedical Sciences, Ulster University, Coleraines BT52 1SA, UK; c.logue@ulster.ac.uk; 11 Research Department of Metabolism and Nutrition, Medical School, Marist University of Merida, Mérida Yucatan 97300, Mexico; hlaviada@marista.edu.mx; 12 Argentine Association of Food Technologists, Buenos Aires 1088, Argentina; tecnologos@alimentos.org.ar; 13 Mexican College of Internal Medicine, Mexico City 03910, Mexico; doctoraldretej@hotmail.com (J.A.A.-V.); dr-emeneses@hotmail.com (E.M.S.); 14 Hospital General de Especialidades del ISSSTE, Saltillo, Coahuila 25020, Mexico; 15 Logre International Food Science Consulting, Mexico City 14650, Mexico; rebecalg@prodigy.net.mx; 16 Clinical Nutrition Department, La Paz University Hospital, Madrid 28046, Spain; cgcandela@salud.madrid.org; 17 Hospital La Paz Health Research Institute - IdiPAZ, Autonomous University of Madrid, Madrid 28046, Spain; 18 Atelier de Nutrição, Lisboa 1250, Portugal; rodrigo@saber-comer.com; 19 Nuclear Cardiology Department, Instituto Nacional de Cardiología “Ignacio Chávez, Tlalpan, Ciudad de Mexico 14080, Mexico; alexandersonerick@gmail.com; 20 Phisiology Department, National Autonomous University of Mexico, Coyoacán, Ciudad de México 04510, Mexico; 21 Mexican Society of Cardiology, México City 14080, Mexico; rolandojoel@gmail.com; 22 Dr. Negrin University Hospital of Gran Canaria, Las Palmas de Gran Canaria 35010, Spain; 23 Department of Pharmacology and Toxicology, Faculty of Veterinary Medicine, Complutense University of Madrid, Madrid 28040, Spain; anadon@vet.ucm.es; 24 Nutritional Epidemiology Unit, University of Paris 13, Bobigny 93017, France; f.bellisle@uren.smbh.univ-paris13.fr; 25 Mexican College of Nutrition - Yucatan, Mérida 97133, Mexico; cincanutricionintegral@hotmail.com; 26 Regional Center for Sports Medicine of the Junta de Castilla y León, Valladolid 47011, Spain; raquelblasco92@hotmail.com; 27 Facultad Mexicana de Medicina, La Salle University, Mexico 14000, D. F., Mexico; ndt@prodigy.net.mx; 28 Mexican Transplant Institute, Cuernavaca, Morelos 62448, Mexico; 29 Serviço de Endocrinologia, Hospital de Santa Maria–CHLN, Lisboa 1649-035, Portugal; jose.camolas@gmail.com; 30 Argentine Quality Institute-Instituto Argentino para la Calidad (IAPC), Ciudad Autónoma de Buenos Aires 1406, Argentina; fgcardini@gmail.com; 31 Mountain Research Centre (CIMO), Polytechnic Institute of Bragança, Campus de Santa Apolónia, Bragança 5300-253, Portugal; mcarocho@ipb.pt; 32 ASAE-Autoridade de Segurança Alimentar e Económica, CBIOS/ECTS-The Biosciences Research Center and NICiTeS/ERISA–Núcleo de Investigação em Ciências e Tecnologias da Saúde, Grupo Lusófona, Lisboa 1749-024, Portugal; p1658@ulusofona.pt; 33 Center for Public Health Nutrition, University of Washington-Center for Obesity Research, Seattle, WA 98195, USA; adamdrew@uw.edu; 34 Chilean College of Nutritionists, Universidad San Sebastian, Santiago 7500000, Chile; sduran74@gmail.com; 35 Nutrition and Food Technology Institute, University of Chile, Santiago 7830490, Chile; vfaundes@inta.uchile.cl; 36 Peruvian Nutrition Society-Sociedad Peruana de Nutrición (SOPENUT), Miraflores 15074, Peru; presidentasopenut@gmail.com; 37 Andalusian Society of Endocrinology, Diabetes and Nutrition, Department of Endocrinology and Nutrition, Virgen del Rocio Hospital, Sevilla 41013, Spain; garcialunapp@yahoo.es; 38 Mexican Nutrition and Endocrinology Society, Mexico City 04330, Mexico; endocrinogarnica@hotmail.com (J.C.G.); agustinmadero@hotmail.com (M.A.M.); 39 ImFINE Research Group, Department of Health and Human Performance, Faculty of Physical Activity and Sport Science-INEF, Universidad Politécnica de Madrid, Madrid 28040, Spain; 40 Department of Clinical Sciences and Community Health, Università degli Studi di Milano, Milano 20133, Italy; carlo.lavecchia@unimi.it; 41 School of Medicine and Dentistry, University of Santiago de Compostela, Santiago de Compostela 15782, Spain; 42 Gastroenterology, Hepatology and Paediatric Nutrition Unit of the Santiago Clinical University Hospital, Santiago de Compostela 15706, Spain; 43 Department of Nutrition and Food Science, School of Pharmacy, Complutense University of Madrid (UCM), Madrid 28040, Spain; asobaler@ucm.es; 44 Spanish Federation of Nutrition, Food and Dietetics Societies, Madrid 28918, Spain; amarcos@ictan.csic.es; 45 Institute of Food Science, Technology and Nutrition (ICTAN); Spanish National Research Council (CSIC), Madrid 28040, Spain; 46 Mexican Nephrological Research Institute, Mexico City 14080, Mexico; mariscalmd@yahoo.com.mx (L.A.M.R.); janc1999@gmail.com (J.A.N.-C.); 47 Intertek, HERS, Cody Technology Park, Farnborough GU14 0LX, UK; danika.martyn@intertek.com; 48 Council for Agricultural Research and Economics, Research Centre for Food and Nutrition, Rome 00178, Italy; lorenza.mistura@crea.gov.it; 49 Department of Food Science and Technology, University of Cordoba, Cordoba 14071, Spain; rafael.moreno@uco.es; 50 Pediatric Department, Cínica Universidad de Navarra, Madrid 28027, Spain; jmorenov@unav.es; 51 Departamento de Nefrología y Metabolismo Mineral, Instituto Nacional de Ciencias Médicas y Nutrición Salvador Zubirán, Tlalpan-Ciudad de México 14080, Mexico; 52 University of Porto, Faculty of Pharmacy, REQUIMTE/LAQV, Porto 4050-313, Portugal; beatoliv@ff.up.pt; 53 Department of Medicine, Endocrinology and Nutrition, Sport Medicine Center, AEPSAD, High Sports Council, Madrid 28040, Spain; nieves.palacios@aepsad.gob.es; 54 Nutriguía Uruguay, Montevideo 11000, Uruguay; nutriguia@nutriguia.com.uy; 55 Departamento de Educación Médica, Instituto Nacional de Ciencias Médicas y Nutrición Salvador Zubirán, Tlalpan-Ciudad de México 14080, Mexico; rinconpedrero@gmail.com; 56 Endocrinology and Nutrition Department, Fundación Jiménez Díaz Hospital, IDC Salud, Madrid 28040, Spain; priobo@telefonica.net; 57 Facultad de Medicina, Universidad Autónoma de Madrid, Ciudad Universitaria de Cantoblanco, Madrid 28049, Spain; 58 Servicio de Gastroenterología, Hepatología y Nutrición del Instituto Nacional de Salud del Niño, Lima 15083, Peru; juan.riveramedina@gmail.com; 59 Departamento de Pediatría, Universidad Nacional Mayor de San Marcos, Lima 15083, Peru; 60 Faculty of Medicine, Autonomous University of the State of Mexico, Toluca 50180, Mexico; rvaldesr@uaemex.mx; 61 Instituto Nacional de Saúde Doutor Ricardo Jorge, Lisboa 1649-016, Portugal; elsa.vasco@insa.min-saude.pt; 62 Nutrition and Public Health Working Group, Argentine Nutrition Society, La Plata, Buenos Aires 1900, Argentina; sandrawac@hotmail.com; 63 Mexican Society of Paediatrics, Mexico City 06760, Mexico; guillewakida@yahoo.com.mx; 64 Scientific and Educational Committee, Spanish Society of Parenteral and Enteral Nutrition (SENPE), Barcelona 08017, Spain; carminaw@telefonica.net; 65 Instituto Nacional de Pediatria, Sociedad Mexicana de Pediatria, Insurgentes Cuicuilco, Ciudad de México 04530, Mexico; xochiludidr@hotmail.com; 66 Mexican Diabetes Federation, Mexico. Facultad de Medicina y Hospital Universitario de la Universidad Autónoma de Nuevo León, Monterrey 64630, Mexico; sergiozungua@gmail.com; 67 International Sweeteners Association-ISA, Brussels 1040, Belgium; scientificdirector@sweeteners.org; 68 Pediatric Hospital of Coimbra-Centro Hospitalar e Universitário de Coimbra, Coimbra 3000-075, Portugal; scvelho@hotmail.com

**Keywords:** non-nutritive sweeteners, low-calorie sweeteners, sweeteners, Ibero-American, safety, regulation, obesity, diabetes, cancer, added sugars

## Abstract

International scientific experts in food, nutrition, dietetics, endocrinology, physical activity, paediatrics, nursing, toxicology and public health met in Lisbon on 2–4 July 2017 to develop a Consensus on the use of low- and no-calorie sweeteners (LNCS) as substitutes for sugars and other caloric sweeteners. LNCS are food additives that are broadly used as sugar substitutes to sweeten foods and beverages with the addition of fewer or no calories. They are also used in medicines, health-care products, such as toothpaste, and food supplements. The goal of this Consensus was to provide a useful, evidence-based, point of reference to assist in efforts to reduce free sugars consumption in line with current international public health recommendations. Participating experts in the Lisbon Consensus analysed and evaluated the evidence in relation to the role of LNCS in food safety, their regulation and the nutritional and dietary aspects of their use in foods and beverages. The conclusions of this Consensus were: (1) LNCS are some of the most extensively evaluated dietary constituents, and their safety has been reviewed and confirmed by regulatory bodies globally including the World Health Organisation, the US Food and Drug Administration and the European Food Safety Authority; (2) Consumer education, which is based on the most robust scientific evidence and regulatory processes, on the use of products containing LNCS should be strengthened in a comprehensive and objective way; (3) The use of LNCS in weight reduction programmes that involve replacing caloric sweeteners with LNCS in the context of structured diet plans may favour sustainable weight reduction. Furthermore, their use in diabetes management programmes may contribute to a better glycaemic control in patients, albeit with modest results. LNCS also provide dental health benefits when used in place of free sugars; (4) It is proposed that foods and beverages with LNCS could be included in dietary guidelines as alternative options to products sweetened with free sugars; (5) Continued education of health professionals is required, since they are a key source of information on issues related to food and health for both the general population and patients. With this in mind, the publication of position statements and consensus documents in the academic literature are extremely desirable.

## 1. Introduction: Justification and Need of the Ibero–American Consensus

Low- and no-calorie sweeteners (LNCS) are food additives that are added to a variety of foods and beverages in place of sugars either during the manufacturing process or as table-top sweeteners. LCNS are primarily used in products because they can provide a desired sweet taste with little or no additional energy; furthermore, they do not elicit the same metabolic responses to sugars and are non-cariogenic. Given these favourable characteristics, LNCS-containing products are often recommended to those individuals living with specific health conditions to improve quality of life by offering reformulated, yet palatable products that are better suited to their health needs (e.g., sugar-free foods and beverages for individuals living with diabetes) [1]. Harmonized legislation on LNCS in foodstuffs was first adopted in the European Union (EU) in 1994 and included maximum permitted levels of each LNCS in specific food categories [2]. According to Regulation (EU) No. 1333/2008 (and amendment 497/2014) of the European Parliament and of the Council of 16 December 2008 on food additives [3,4], all food additives must be subject to a safety evaluation by the European Food Safety Authority (EFSA) before they are permitted for use in the EU. As defined by Regulation (EU) No 257/2010 [5], a programme of re-evaluation on food additives that were approved for use in EU before 20 January 2009 must be conducted by EFSA. For LCNS, this re-evaluation will be concluded by the end of 2020.

Despite comprehensive safety evaluations by regulatory authorities, LNCS are often associated with a range of adverse health outcomes. For example, the role of LNCS on cancer risk has been widely debated since the 1970s following observations of increased bladder cancer risk in rodents treated with extremely high doses of saccharin [6]. However, earlier epidemiological studies in humans found inconsistent associations with bladder cancer risk. Furthermore, the proposed associations were not confirmed in subsequent studies, and mechanistic data showed different saccharin metabolism in rodents and humans. The mechanism for saccharin-induced bladder cancer has been hypothesized to involve the binding of saccharin to urinary proteins, initiating the subsequent formation of silicate-containing precipitate and crystals; the urinary crystals act as an abrasive to the bladder epithelium, causing cytotoxicity with resultant regenerative hyperplasia [7]. This putative mechanism is not relevant to humans so for this reason, and the fact that no clear evidence that saccharin is carcinogenic in humans exists [8,9], saccharin was delisted in 2000 from the U.S. National Toxicology Program’s Report on Carcinogens, where it had been listed since 1981 as a substance reasonably anticipated to be a human carcinogen. The information about the delisting of saccharin is available in the Report on Carcinogens, Fourteenth Edition (2016) of the U.S. Department of Health and Human Services [10].

Other concerns include a potential role for LNCS in food intake, mood, blood pressure, body weight and abdominal obesity, diabetes, dental caries, neurodegenerative diseases or dementia; however, the evidence available to date on those outcomes is inconsistent [11].

Some controversy exists regarding consumption of LNCS during pregnancy and in young children. A position statement from the US Academy of Nutrition and Dietetics supported the assertion that consumption of LNCS within stated acceptable daily intakes (ADI) is safe in pregnant woman and in young children [12,13,14]. However, that position is currently under revision and the Institutes of Medicine (IoM) argue that there is a lack of evidence on the long-term health effects of the use of LNCS when used from early childhood [15]. It is important to clarify that a comprehensive toxicological evaluation is conducted for all LNCS prior to approval at national and international levels, which considers reproductive toxicology and exposure during pregnancy and early life [16]. Furthermore, recently published literature reviews on the health impacts of LNCS use in early life have highlighted apparent gaps in current knowledge and the requirement for further work in the area [17].

Several decades ago, there were relatively few ingredients available to sweeten foods and beverages; however, there are now dozens that can be used to replace sugar in products. Therefore, ensuring consumer understanding and awareness of the regulatory processes is of utmost importance to ensure appropriate use of LNCS. Indeed, consumption of LNCS has increased over the past 30 years and with that, consumer concerns about their safe use [18]. It is also worth noting that many foods, even those that do not claim to be sugar-free, may contain LNCS. Although LNCS may be present in foods and beverages that do not claim to be sugar-free or free of added sugars, labelling regulations are consistent in most countries globally (including all Ibero–American countries) in that the presence of LNCS in products must be declared on the list of ingredients. To assist consumers, there is a legal requirement in all countries to label the ingredients used in a food or beverage placed on the market (in the EU for example, the presence of a sweetener in a food must be labelled twice). The debate on labelling regulations on sugar and LNCS is increasing at different levels, and not always based on scientific evidence.

Based on this premise, and as a continuation of a previous multidisciplinary meeting of experts in LNCS [19], 66 international scientific experts in food, nutrition, dietetics, endocrinology, physical activity, paediatrics, nursing, toxicology and public health met in Lisbon on 2–4 July 2017 to develop a consensus on the use of LNCS as substitutes for sugar and other caloric sweeteners. The event was organised by the Spanish Nutritional Research Foundation (FIN) in collaboration of the Lusófona University of Lisbon, and with the support of 43 organisations and foundations specialised in nutrition and dietetics, medical societies, universities and research centres in Europe and Latin America.

The experts of the Lisbon Consensus analysed and evaluated the role of LNCS in the diet, their safety and regulation, and the nutritional and dietary aspects of their use in foods and beverages. The goal of this Consensus document was to provide a useful, evidence-based, point of reference to assist in efforts to reduce free sugars consumption in line with current international public health recommendations [20], in the context of the prevention and treatment of obesity and related diseases in Latin American countries. Speakers were asked to include high quality and independent systematic reviews in their presentations during the meeting. These results were discussed on the basis of its results, methodological quality and policy implications.

## 2. Safety of LNCS and Monitoring Systems

### 2.1. Background

The safety of approved LNCS have been repeatedly assessed and confirmed by numerous risk assessment regulatory and scientific bodies. Indeed, LNCS are the most extensively researched food additives available on the marked. Although all LNCS induce perceptions of sweetness, they are chemically diverse with varied kinetics, i.e., absorption profiles, metabolic fates and excretion pathways [21]. An often unrecognized aspect of their safety profile is that establishing these characteristics forms a critical part of their safety assessment [22].

A risk assessment comprises hazard identification, hazard characterisation, exposure assessment and risk characterisation. A long process of scientific risk assessment is required before the technical review of food additives [23], and numerous national and international scientific and regulatory bodies devoted to risk assessment undertake this process prior to the approval of LNCS. Regulatory bodies such as the US FDA (Food and Drug Administration), the EFSA (European Food Safety Authority, not completely regulatory) or ANMAT (Argentina National Administration of Drugs, Food and Medical Technology) require data on reproductive and developmental toxicity, as well as mutagenicity/genotoxicity, carcinogenicity, immunotoxicity neurotoxicity, from a battery of acute and chronic studies before a food additive can be considered for use.

### 2.2. International Regulatory Bodies

International organisations that intend to harmonize the use of LNCS include the Joint Food and Agriculture Organisation/World Health Organisation (FAO/WHO) Expert Committee on Food Additives (JECFA) and the Codex Alimentarius Commission (CAC). The objective of the FAO/WHO program on food additives is to conduct systematic safety evaluations and provide advice to Member States about the control of food additives, as well as the related health aspects of their use. The JECFA and the CAC are the two bodies responsible for implementing the program [16].

The JECFA is an international group of scientific experts who serve in their personal capacities rather than as representatives of their governments or other institutions. Their reports contain the collective views of the group of experts and do not necessarily represent the decision, or the stated policy, of the WHO or FAO. The experts convene to provide advice on technical and scientific matters, establishing specifications for the identity and purity of food additives, evaluating the toxicological data, and recommending, where appropriate, ADI levels for humans. The committee also acts in an advisory capacity for the Codex Committee on Food Additives (CCFA) and the Codex Committee on Contaminants in Food [16].

The CAC was established in 1962 to implement the Joint FAO/WHO Food Standards Program; Codex members cover 99% of the world’s population. The Codex Alimentarius is a collection of international food standards, guidelines and codes of practice that aim to protect consumers’ health and contribute to the safety, quality and fairness of international food trade [24]. The Codex Alimentarius also includes provisions for food additives. The CCFA is charged with establishing or endorsing acceptable maximum levels for individual food additives, preparing priority lists of food additives for risk assessment by the JECFA, assigning functional classes to individual food additives, recommending specifications for identity and purity of food additives for adoption by the Commission, considering methods of analysis for the determination of additives in food, and considering and elaborating standards or codes for related subjects such as the labelling of food additives when sold as such [24].

The CCFA has developed a General Standard for Food Additives (GSFA) online database [25] that lists food additives reviewed and assigned an ADI (either numerical or “not specified”) by the JECFA and provides all the provisions for food additives that have been adopted by the CAC. The GSFA provides a list of food categories for which an additive may be used and the levels of use for each food category [25]. Countries that do not have the review capability rely on JECFA for scientific evaluation of food additives and the categories of use and use level guidelines provided by the CCFA in the GSFA.

The World Trade Organisation (WTO) encourages countries to harmonize food standards based on Codex standards and uses its decisions to settle trade disputes. In addition, the WTO recognises JECFA specifications for food additives in international trade, increasing the importance of both the Codex and JECFA.

At the European level, EFSA was created in January 2002 as an independent source of scientific advice and communication on risks associated with the food chain. EFSA’s Scientific Committee and Panels carry out EFSA’s scientific risk assessment work. LNCS issues are addressed by EFSA’s Panel on Food Additives and Nutrient Sources Added to Food (ANS) [26,27].

In the EU, the harmonization of legislation to ensure that all Member States have similar laws and regulations is an ongoing process. The European Parliament and the Council adopted a framework regulation (Regulations 1333/2008 and 497/2014) [3,4], which consolidates all current authorisations for food additives, including LNCS, into one legal text, as of January 2011 [28].

The following LNCS are authorised in the EU: acesulfame K, advantame, aspartame, cyclamates, neohesperidine DC, neotame, saccharins, salt of aspartame-acesulfame, steviol glycosides, sucralose and thaumatin (see Table 1).

In accordance with the above regulations, EFSA has started a systematic re-evaluation of authorised food additives including LNCS, and is issuing scientific opinions according to the priorities indicated in the Regulation (EU) No 257/2010. To ensure an effective re-evaluation, EFSA retrieves relevant data from interested parties for the re-evaluation of the selected food additives by launching public calls for data to acquire documented information (published, unpublished or newly generated) on technical and toxicological data on LNCS authorised as food additives in the EU.

In the United States of America, the majority of the modern-day LNCS: acesulfame K, advantame, aspartame, neotame and sucralose have been approved through the food additive process [37], whereas the most recent LNCS approvals for steviol glycosides and lo han guo have occurred through the Generally Recognised as Safe (GRAS) system [38], based on scientific procedures. While the regulatory process and review time of these two types of evaluations by the US FDA differ, the same level of scientific evidence is required to support safety to ensure reasonable certainty of no harm [39].

Regulatory approvals in other jurisdictions follow different paths and these will be discussed in subsequent sections.

The resulting food safety frameworks, as well as toxicological assessments driving these frameworks, rely in many cases on extrapolations from animal studies to humans and use endpoint-based No Observable Adverse Effect Levels (NOAEL) to derive health-based guidance values (HBGV) such as ADI. For most chemical compounds, hazard characterisation is based on estimation of an intake for humans that would be below the dose necessary to produce adverse effects. Uncertainty (safety) factors are used to convert a surrogate for the threshold, such as the NOAEL in animals into a “safe” intake for humans.

### 2.3. Acceptable Daily Intake

Another important part of the scientific risk assessment of food additives is linked to the notion of the dose-response relationship and the determination of the ADI (i.e., hazard characterisation of scientific risk assessment). JECFA defines the ADI “for man, expressed on a body weight basis, is the amount of a food additive that can be taken daily in the diet, even over a lifetime, without risk” [16,23]. The ADI is expressed in milligrams per kilogram of body weight per day.

The ADI is a conservative estimate that incorporates a considerable safety factor. It is established from toxicological testing in animals [40], and sometimes humans, and is usually established by applying an intentionally conservative safety factor (generally a 100-fold safety factor) to the NOAEL. Animal tests are used to determine the NOAEL by identifying the maximum dose of an additive that results in no toxic effects. A safety factor of 100 is subsequently used, which comprises two 10-fold factors that account for inter- and intra-species variability. For example, if safety evaluation studies of a given substance demonstrate a NOAEL of 1000 mg/kg body weight per day, using a 100-fold safety factor the ADI would be 10 mg/kg body weight per day for humans.

The ADI does not represent a maximum allowable daily intake level. It should not be regarded as a specific point at which safety ends and possible health concerns begin. In fact, the US FDA has said it is not concerned that consumption occasionally may exceed the ADI. The FDA agency has stressed that because the ADI has a built-in safety margin and is based on a chronic lifetime exposure, occasional consumption in amounts greater than the ADI “would not cause adverse effects” [41].

Furthermore, the projected consumption of potentially high consumers of a certain food in which the additive will be used are considered during the risk assessment process [23]. Risk assessment, including dietary exposure assessment, provides the scientific basis for the establishment of standards, guidelines and other recommendations of the CAC. This ensures that safety requirements for food are protective of public health, consistent between countries and appropriate for use in international trade.

Despite this intensive scientific risk assessment, deciding on the absolute safety of a certain food additive is not possible, as scientific practice is usually linked to a level of uncertainty [23,42].

Not all LNCS are assigned an ADI following safety and toxicological assessments. The ADI ‘not specified’, which is used to refer to a food substance of very low toxicity based on the available data (chemical, biochemical, toxicological and other) and the likely total dietary intake of the substance at the proposed levels of use, does not, in the opinion of the Committee, represent a hazard to health. For that reason, and for the reasons stated in the individual evaluations, the establishment of an ADI expressed in numerical form is not deemed necessary. However, a food additive that meets this criterion must still be used within the bounds of good manufacturing practice, i.e., it should be technologically efficacious and should be used at the lowest level necessary to achieve its effect, it should not conceal food of inferior quality or adulterated food, and it should not create a nutritional imbalance [16,43].

It is preferable to establish guideline values for exposure limits based on health criteria that cover the entire population. These values are usually set to protect the most vulnerable subpopulation, based on critical health outcomes in the most susceptible. If this is the case, following the recommendation to establish a single ADI value, the most conservative data will be taken for the most sensitive individual of the population [16,43].

In dietary exposure assessments of chemical compounds in food, data on food consumption are combined with the concentration of chemical compounds in food. The resulting dietary exposure estimate can then be checked against the guideline value for exposure limits based on health criteria or with the toxicological point of departure (NOAEL) for the chemical compound as part of the characterisation of the risk. A tiered approach is often implemented beginning with conservative estimates with more refined, and costly, methodologies indicated if intakes are deemed to exceed the ADI. Acute or chronic exposure may be determined. Food exposure determinations include the general population as well as vulnerable groups or where exposure is expected to be significantly different from that of the general population (i.e., infants, children, pregnant women, the elderly, diabetics, and vegetarians). Information on food consumption is derived from specific consumption data for the local population, which include national food consumption survey datasets, as well as the amounts of food for human consumption available from the statistics of production, disappearance or use of food [16,43].

### 2.4. Regulation at National Level

#### 2.4.1. Mercosur

The ministries of Health, Agroindustry and Productions (with variations according to the countries) and national administrations of each member country of MERCOSUR are the competent agencies that implement this regulation (Argentina: National Administration of Drugs, Food and Medical Technology—ANMAT; Brazil: National Health Surveillance Agency—ANVISA; Paraguay: National Institute of Food and Nutrition—INAN; Uruguay: Technological Laboratory of Uruguay—LATU).

The member countries of MERCOSUR regulate the use of LNCS through the MERCOSUR Technical Regulation: Harmonised General List of Food Additives and Their Functional Classes, MERCOSUR/GMC/RES. No. 11/06 [44].

The following LNCS are authorised in the MERCOSUR: acesulfame K, aspartame, cyclamate and its salts (Na, K, Ca), saccharin and its salts (Na, K, Ca), neohesperidine DC, neotame, sucralose, thaumatin and steviol glycosides.

#### 2.4.2. Chile

The Ministry of Health regulates the use of LNCS through the Sanitary Regulation of Foods (RSA) Decree. No. 977/96 of the Ministry of Health, Article 146, indicating that only non-nutritive sweeteners, as indicated in this article (acesulfame K, aspartame, cyclamate and its Na and Ca salts, saccharin and its Na and Ca salts, sucralose and steviol glycosides), are permitted in foods for weight control regimes and in foods with low fat or energy content. The RSA uses the ADI values recommended by FAO/WHO for each LNCS and in Chile it is mandatory to state the amount of each LNCS used in food and beverages on the labels along with the respective ADI [45].

#### 2.4.3. México

LNCS that have been authorised and assigned an ADI are listed in the agreement that determines the use of food additives and coadjutants in foods, beverages and food supplements as approved by the Secretary of Health of the United States of México, dated 16 July 2012 [46]. The most recent update of this document was on 18 October 2017 [47]. This list includes: sucralose, saccharin, neohesperidine DC, neotame, steviol glycosides, aspartame, aspartame-acesulfame, advantame, alitame, acesulfame K, cyclamates and allulose (recently moved to listing in Annex VIII LNCS that can be used according to Good Manufacturing Practices). Regarding labelling rules, table-top sweeteners, whatever their form of presentation, should indicate the concentration per serving and their corresponding ADI.

#### 2.4.4. Ecuador

The Ecuadorian technical standard, NTE INEN 074-2012 lists approved food additives for human consumption, the positive lists and their requirements.

In its introduction, the Technical Subcommittee (TSC) of Additives approved the adoption of the document Codex Stan192-1995 Codex General Standard for Food Additives as the NTE INEN 2074—second revision and establishes that it should be immediately updated without the need to reconvene the TSC as the Codex document is revised or amended [48]. In addition, note 1 establishes that the use of food additives not taken into consideration in the NTE INEN 2074 will always be allowed, if it is demonstrated that there is an authorisation of use in any CFR 21 by FDA or if its use as a food additive is incorporated by a Directive of the EU.

The Ecuadorian Technical Regulation RTE INEN 022 (1R) “Labelling of processed and packaged food products”, defines non-caloric sweeteners as any natural or artificial substance used to sweeten and that does not provide energy. It establishes in its article 5.5.9 that the food products that contain among their ingredients one or several non-caloric sweeteners, must include on their labels the message: “This product contains a non-caloric sweetener”.

#### 2.4.5. Peru

In Peru, the Supreme Decree No. 007-98-SA, Regulation on the Surveillance and Sanitary Control of Food and Beverages, clearly establishes in Article 63 that the use of food additives that are not included in the list of additives permitted by the Codex Alimentarius are prohibited. In addition, food additives that are accepted by the US FDA, the EU and the Flavour and Extractive Manufacturing Association (FEMA) are also permitted. There are reference technical norms available of voluntary use. The NTP 209.703:2012 lists the permitted LNCS, as well as the polyols admitted in Peru [49].

#### 2.4.6. Canada

In Canada, food additives are regulated under the Food and Drug Regulations and associated Marketing Authorisations (MAs). All permitted food additives and their conditions of use are listed in the Lists of Permitted Food Additives [50]. The Food and Drug Regulations (the Regulations) require that food additives meet certain standards for identity and purity for the additive to be considered food-grade. These standards, or specifications, were updated in the Regulations on 14 December 2016 [50].

In Canada, food additives such as sugar substitutes, which cover both artificial and intense sweeteners obtained from natural sources, are subject to rigorous controls under the Food and Drugs Act and Regulations. This List of Permitted Sweeteners sets out authorised food additives that are used to impart a sweet taste to a food. It is incorporated by reference in the MAs for Food Additives that may be used as Sweeteners. The Maximum Level of Use is expressed in ppm (mg/kg food) or % in products as consumed. The following LNCS are listed: advantame, acesulfame K, aspartame, saccharin and its salts (Na, K, Ca), neotame, steviol glycosides, sucralose, monk fruit extract and thaumatin [50].

### 2.5 Key Points: Safety of LNCS and Monitoring Systems

- LNCS are some of the most extensively evaluated dietary substances.
- The safety of approved compounds is proven and is continuously re-evaluated to consider new and relevant scientific data.
- The safety of LNCS such as aspartame, cyclamate and saccharin has been reviewed and re-affirmed several times by the US FDA and by EFSA, who recently completed a full risk assessment of aspartame [51].
- Since taste and food application profiles among LNCS can vary, the use of LNCS blends can be important for developing lower-sugar finished food products.

## 3. Food Composition and Nutrition Labelling for LNCS

### 3.1. Background

LNCS provide a desired sweet taste with the addition of little or no energy. They can be added to a wide range of sugar-reduced or sugar-free products such as soft drinks, chewing gum, confectionery, frozen desserts, dessert mixes, yogurts and puddings depending on the approved conditions of use.

In the EU, food labelling related to food additives must meet the requirements laid out in Regulation (EU) No. 1169/2011 of the European Parliament and of the Council of 25 October 2011 [52] on the provision of food information to consumers. According to EU labelling regulations, the presence of an LNCS in foods and beverages must be labelled twice on food products; the name of the LNCS (e.g., saccharin) or the E-number (e.g., E954) must be included in the list of ingredients, and, the term ‘with sweetener(s)’ must also be clearly stated on the label together with the name of the food or beverage product. Therefore, it is intended that consumers are always duly informed when a food (including beverage) product contains an LNCS; however, consumers may not always recognise this information, particularly with existing concerns that food labels often contain large amounts of information that may leave some consumers feeling overwhelmed. Separately, individuals living with phenylketonuria (PKU) require a strict adherence to a low phenylalanine diet and aspartame is a known, but not only, source of phenylalanine in the diet [51].

Therefore when a food or beverage contains aspartame or aspartame-acesulfame salts, the product label must bear the phrase, “contains a source of Phenylalanine”.

In other countries such as Chile, the Sanitary Food Regulation [45] stipulates that labelling must not only clearly indicate the LNCS being used, but should also indicate the concentration of LNCS per of 100 g or 100 mL of the product. Furthermore, the ADI (expressed in milligrams per kilogram of body weight per day) for each LNCS used in the product, as set by FAO/WHO, should also be included. In the case of aspartame, it must be indicated on the labelling; “Phenylketonurics: contains phenylalanine”.

In MERCOSUR countries, labelling regulations are governed by Joint Resolution No. 149/05 SPRRS and No. 683/05 SAGPyA, which incorporates Resolution GMC 26/03: MERCOSUR Technical Regulation for the Labelling of Packaged Foods and Resolutions GMC 44/03 and 46/03: MERCOSUR Technical Regulations for the Nutritional Labelling of Packaged Foods. Foods containing LNCS must record the concentrations of LNCS used in mg/100 g or 100 mL, with characters of a size not less than 1.5 mm in height.

Nutrition research needs much more comprehensive labelling databases that can capture information on newly introduced or reformulated products [53,54]. It is important to clarify that labels follow regulatory requirements and LNCS are listed on the ingredient list according to the requirements in each country. Exposure data is considered when each LNCS is approved in each country or market (for the cases of the EU and Mercosur). On the other hand, important food composition databases are not updated frequently enough to show the rapidly occurring changes in the food supply and market [55].

Therefore, to identify the main sources of LNCS according to gender, age, or socioeconomical status, these factors should be considered in the design of future intake studies. It is important to specify that prior to approval of each LNCS, and as part of the risk assessment process, potential exposure is evaluated through the estimated daily intake of the most vulnerable subpopulation. This is calculated to ensure that the levels of exposure stay within the ADI [16]. There are several recently published studies that have assessed the dietary intake of LNCS in different populations in Europe and in other regions of the world which conclude that the intake of LNCS is well below the ADI value even for high consumers [56,57,58,59].

Campaigns aiming to help consumers “to read and understand” food labels must be encouraged, particularly in reformulated products where calories or sugar are reduced. To maximise the effectiveness of manufacturing strategies aimed at making products healthier, it is essential to ensure some level of consumer understanding of the regulations governing such matters. For example, adequate understanding of the legal definition of terms such as “free”, “reduced”, “low” may help consumers make informed choices; however, it is worth noting that this terminology is not harmonised throughout the countries.

Most consumers are unaware that foods and beverages may contain ingredients that counterbalance sweetness (e.g., the sweet-taste receptor antagonist lactisole (sodium 2-(4-methoxyphenoxy) propanoate). Thus, even the most health-conscious individuals, including parents may not understand the information provided in the ingredient lists and may rely instead on short and uncomplicated nutrient-content claims for guidance [60].

The US FDA requires that the ingredient lists of all LNCS-containing products include the specific LNCS name, but the amount that has been added to the product remains proprietary information. Thus, even if motivated individuals have explored the ADI value of a particular LNCS, they cannot determine how much of a beverage containing that LNCS can be consumed to ensure intakes remain within the ADI [60].

In other countries, LNCS content should also be declared. In Canada in addition to the requirement to label on the front of a food or beverage package that it contains one or more non-nutritive sweeteners (e.g., “contains aspartame”), the amount of the sweetener or sweeteners expressed in milligrams per serving and a statement describing the sweetness per serving, expressed as the amount of sugar needed to produce an equivalent degree of sweetness, must also be present on the package and grouped with the ingredient list [61]. Similarly, as described in the previous section, several South American countries mandate the labelling of the level of LCNS on the food label, including the ADI, for any sweeteners present.

In general, food label and nutrient composition databases (Food Composition Tables (FCT)) could assist with understanding patterns of intake, particularly FCTs that are regularly updated to incorporate new and newly reformulated products introduced into the market [62]. Since 2005, EU food manufacturers are required to provide EFSA detailed information on the quantity of food additives used in the foods and beverages on the EU market as part of EFSA “Calls for data”. However, as noted above, food and beverage manufacturers are not always required to show the content of these substances on food labels, particularly globally, which makes it difficult to obtain precise information about overall intake.

### 3.2. FCT and LNCS

There is a growing interest in studies estimating intake of LNCS, mainly in relation to possible changes in dietary patterns in populations [17]. Recently published studies using analytical dietary intake assessment methods show that the consumption of LNCS is well below the ADI even for high consumers in different European populations [56,57,58,59].

LNCS are not identified in some FCT as a possible component of a food or beverage, nor are the levels of LNCS identified in food label databases. Consequently, it is often difficult for one to accurately establish the amount and type of LNCS that are being consumed within the overall diet. As such, it is proposed that LNCS should appear annexed to the FCT as this information would be a valuable addition to the available data on the amounts and types of LCNS in various foods.

To this end, efforts are urgently needed to collaborate with the food industry to obtain updated information about composition of foods and beverages, which would allow adapting FCT at national level in a first stage. In Europe, the EFSA collect this type of information from manufacturers.

It was agreed that there can be significant regional and national differences in the LNCS content of products, even for a “similar” product and/or brand and therefore, complete and updated FCT at this level must be encouraged.

When reviewing the available scientific information from the studies assessing the LNCS, misinformation/misreporting are common. In consequence, efforts should be made to have FCTs updated to include LNCS, and outcomes validated.

### 3.3. Key Points: Food Composition and Nutrition Labelling for LNCS

- It seems necessary to prepare a comparison table for the Ibero–American countries to include the different labelling regulations at present, highlighting strengths and weaknesses.
- It seems critical and urgent to unify the terminology used for LNCS, with special focus on the consumer information.
- The labelling must be clear and precise.
- The use of new technologies to expand the information in the printed label is also encouraged for LNCS.
- Consumer education about additive products must be strengthened in a rigorous, objective way, based on the best scientific evidence and regulatory processes.
- Responsible Administrations and Scientific societies should disseminate clear, objective information about LNCS on their websites and social networks and publish educational materials that contribute to the dismissal of doubts and any misinformation that may exist. This may reinforce the information in the labelling and expansion, as well as trust by the consumers.
- Training provided to primary care and specialized healthcare and educational professionals should be made a priority, in order to be able to explain benefits/risks for the LNCS to the consumer, and labelling content.

## 4. LNCS Role in Body Weight Management and in Chronic Diseases

### 4.1. Background

Over recent decades, a growing body of evidence suggesting links between diet, lifestyle, and health has led to public health recommendations to limit or avoid the consumption of foods and beverages high in sugar, salt or fat [63,64,65]. LNCS have been promoted as a possible tool for helping to reduce sugars and overall energy intakes by providing a desired sweet taste with little or no additional energy; however, debate persists around the actual benefits of using LNCS for this purpose [66]. Appetite control is a physiologically complex mechanism influenced by both stimulating factors, including the fact that sweet taste is a powerful mechanism of reward, and inhibitory factors that contribute to limiting intake it each eating occasion and between intakes. A recently published review of animal studies has proposed biologically plausible mechanisms for weight gain and metabolic derangement in LNCS [67]. Nonetheless, the suggestion that LNCS, which provide sweet taste without providing energy, may confound the regulatory mechanisms of appetite and satiety in rats [68] was disproved for humans [69].

LNCS contribute little or no energy [70] as they are either not metabolised following ingestion or due to the small quantities used in food, that their contribution to energy intake is negligible [71,72]. Each LNCS has unique properties in terms of sweetening power (see Table 1), intensity, persistence of sweet taste or aftertaste effect [72]. As such, they are often used as blends to achieve the desired sweetness profile. The human desire for sweet taste embraces all ages, races and cultures; newborn infants exposed to different taste stimuli accept the sweet but reject the bitter taste [73,74]. In the course of evolution, the innate appetite for sweet taste is thought to have constituted a survival advantage, notably by helping to orient food behaviours of newborn mammals towards the intake of nutrient-rich foods [75].

As consumption of foods and beverages sweetened with LNCS has increased, questions about their benefits and possible adverse effects on health have also arisen [76,77]. Some studies have investigated potential short-term effects, for example on food intake, mood or blood pressure [78,79,80]. In other cases, research has focused on long-term effects such as body weight, cancer, diabetes and dental caries. The available evidence is inconsistent and there are still many gaps in knowledge [11]. It has been suggested that the consumption of foods and beverages containing LNCS may be positively associated with overweight and abdominal obesity [67,81]. Some of these effects have been linked to potential alterations in the release of gastrointestinal hormones, gastric motility or alterations of the gut microbiota [82]. These suggested effects are not consistent with the results of several recent randomised controlled trials, which demonstrate that use of an LNCS in place of sugar can help in achieving a reduction in excess body weight [83,84]. Furthermore, there is a lack of evidence for effects of LNCS on gut hormones and function in human studies [85].

A recent study suggested that consumers who choose products labelled “low calorie” may consume sufficient quantities that could eventually lead to an excess of energy intake [86]. In addition, it has also been suggested that the consumption of food and beverages with LNCS may predispose individuals to increased intake of sweet-tasting foods and promote preference for sweetness [87,88]. In a systematic review, Rogers et al. proposed several potential effects of LNCS on body weight, namely (1) disrupt the learned control of energy intake; (2) increase an individual’s desire for sweetness (and thereby cause overeating); or (3) lead to conscious overcompensation for ‘calories saved’ [84]; however, further research is needed to examine the robustness of these hypotheses in real-life scenarios.

Studies which have examined the dietary profile of LNCS consumers have reported higher healthy eating index scores among LNCS consumers compared to non-consumers [89]. In addition, LNCS consumers reported more frequent healthy behaviours, such as refraining from smoking and higher physical activity levels [89,90].

The overall data suggests that the consumption of LNCS does not in itself prevent obesity or directly induce weight loss, but when used in place of sugar, these substances can contribute to weight loss in the short-term and help in the subsequent maintenance [91], as well as improving glycaemic control when used to replace caloric sweeteners. LNCS can therefore be a useful tool in designing food planning and lifestyle for people with overweight, obesity and glycaemic disorders.

The authors of a recent systematic review on the long-term metabolic effects of exposure to LNCS initiated at early ages concluded that the evidence is inconsistent and contradictory, since in some cases they are associated with a possible increase in body mass index (BMI) and accumulation of fat, although it was determined that further research was needed before recommendations could be made [92]. In any case, causal links should be clarified. As discussed below systematic reviews of RCTs indicate that the use of LNCS may help in the reduction of energy intake and thus in weight loss in adults [83,84]. Furthermore, there are no RCTs showing that the use of LNCS when used in place of sugar can lead to weight gain.

With regard to dental health, there is some evidence that the use of toothpaste with fluoride and xylitol may be more effective in preventing tooth decay than the use of toothpaste with fluoride-only in children permanent dentition [93]. The evidence is insufficient to determine whether other products with xylitol could prevent dental caries in infants, children or adults but chewing gums have shown promising results [93]. Other health problems that have been suggested to be associated with the intake of LNCS are neurodegenerative diseases and dementia, although the evidence available in this regard is inconsistent [11].

In 2011, EFSA’s NDA Panel evaluated the substantiation of claims related to intense sweeteners and certain proposed beneficial health effects. The Panel concluded that there is sufficient scientific information to support claims that intense sweeteners lead to a lower rise in blood sugar levels after meals if consumed instead of sugars, and can maintain tooth mineralisation by decreasing tooth demineralisation if consumed instead of sugars. In contrast, the Panel determined that there was no clear cause-and-effect relationship to substantiate the claims that intense sweeteners, when replacing sugars, maintain normal blood sugar levels, or maintain/achieve a normal body weight [28].

### 4.2. Observational Studies

Published systematic reviews of observational studies have concluded that associations between LNCS intake and increased BMI or increased body weight are inconsistent. In a review conducted by Rogers et al. five out of the twelve observational studies reported a positive association between LNCS and higher risk of obesity, while six studies reported a lower risk of obesity and one identified mixed association outcomes between boys and girls [84]. Similarly, in an earlier systematic review and meta-analysis of LNCS intake and weight gain, no statistically significant associations were observed between LNCS consumption and weight gain or increased fat mass [83]. In a subsequent systematic review and meta-analysis by Azad et al., the authors concluded that observational data from prospective cohort studies suggest that routine consumption of LNCS may be associated with a long-term increase in BMI and elevated risk of cardiometabolic disease [77].

An example of an observational study suggesting an association between LNCS and risk of obesity is the San Antonio Heart Study 2008 [94], which reported a positive association between intake of beverages sweetened with LNCS and the incidence of overweight and obesity when participants were followed for a period of 8 years. The variation of the BMI during follow-up was +47% (taking in account the deltas of BMI) reported among consumers of LNCS (+1.48 kg/m^2^) versus non-users (+1.01 kg/m^2^). A similar case is the cohort study “Multi-Ethnic Study of Atherosclerosis” (MESA) of 6814 adults [95]. Daily consumption of soda sweetened with LNCS was associated with a 36% increased risk of metabolic syndrome (HR: 1.36; CI: 1.11–1.66; *p* < 0.001) and also a 67% increase of risk of type 2 diabetes (HR: 1.67; CI: 1.27–2.20). Some observational studies evaluating the relationship between the consumption of beverages sweetened with LNCS and the incidence of metabolic syndrome had reported significant associations even after the adjustment with multivariate analysis. However, many of these studies did not perform an adjustment of variables related to adiposity. When an adjustment was made BMI and waist circumference on crude data in MESA, the association with obesity or diabetes lost statistical significance (HR: 1.17; 95% CI: 0.96–1.44; *p* = 0.06). Associations derived from observational studies must be interpreted with caution, as there are limitations to accurate assessment of the intake of LNCS through surveys and the variation in body weight. In addition, it is difficult to determine the directionality of the effect due to the possibility of “reverse causality”. This implies that subjects who are already obese or overweight (or its risk factors, or genetic predisposition to obesity or diabetes) consume more foods and drinks sweetened with LNCS in order to reduce or mitigate their condition, and not the reverse. In a recent review published by Romo-Romo et al. (2017) [96], prospective cohort studies were condensed. The authors observed an attenuation of the association of LNCS with obesity and metabolic diseases, once the analysis includes adjustments of variables related to adiposity [96]. For this reason, Romo-Romo et al. concluded that “evidence from prospective observational studies indicates that there might be a relationship between the consumption of LNCS sweetened beverages and metabolic disease development”. However, they pointed out that “one possible explanation for the attenuation or loss of the associations after adjusting for adiposity, is that people who consume more LNCS sweetened beverages are more likely to gain weight and therefore tend to consume LNCS as a strategy for weight loss and reduce their energy intake” [96]. In this situation, there could be other genetic, social, family or lifestyle factors that can produce an impact on the development of these diseases rather than the intake of LNCS. In addition, overweight subjects are at a higher risk developing metabolic diseases, there are many studies reporting that overweight people tend to consume more LNCS than lean subjects.

Even though reverse causality does not fully explain higher risks in human [67], studies designed to establish causality are required in order to establish a cause-effect relationship, for example, randomized controlled trials (RCTs) and systematic reviews of RCTs.

Another important limitation of many observational cohort studies is that they often only consider LNCS-containing beverages as the sole source of LNCS; however, these food additives can be found in a wide range of dietary and non-dietary products. Therefore, the self-declared “non-users” can also be exposed to LNCS via the consumption of a range of other products. Furthermore, as previously stated, LNCS represent a chemically diverse group of food additives that undergo different biological fates within the body and therefore future research approaches should aim to distinguish between consumption of individual and combinations of LNCS to more robustly investigate relationships with health. Possible methods of objectively and more comprehensively and specifically assessing consumption of LNCS may be the implementation of a biomarker approach [97].

Taken together, the results from observational studies investigating the relationship between LNCS, body weight and obesity are inconsistent. Observational studies are difficult to interpret as associations may be due to confounding or reverse causality.

In general, LNCS consumption is associated with higher body weight and metabolic disease in observational studies. In contrast, randomized controlled trials demonstrate that LNCS may support weight loss, particularly when used alongside behavioral weight loss support. Additional long-term, well-controlled intervention studies in humans are needed to determine the effects of LNCS on weight, adiposity, and chronic disease under free-living conditions [98].

### 4.3. Intervention Studies

Although controlled clinical trials are the best design for proving cause-and-effect relationships, they tend to be costly, typically have a small sample size and it can be difficult to maintain long-term controlled trial scenario. Furthermore, the use of LNCS in a free-living situation often reflects a ‘choice’ by the consumer and therefore research studies should also be designed to consider this potentially important factor. A review of controlled clinical trials is provided below:

(a) *Short-term RCTs*. Tordoff and Alleva (1990) [99] provided soft drinks sweetened with LNCS or sugar or without any kind of LNCS (controls) to young male and female adults (BMI ≥ 25 kg/m^2^; *n* = 30) during three periods of 21 sequential days. All subjects received any subsequent order of the three modalities. Drinks were to be added to their normal daily diet, and the subjects were instructed to consume four bottles of 300 mL daily. The daily caloric load represented by the soft drinks sweetened with sugar was 530 kcal, with 3 kcal/day provided by soft drink sweetened with LNCS. The participants were instructed to make a daily record of their intake keeping a weekly report of their body weight. Analysis of the information indicated that the group with sugary drinks experienced a small but significant weight gain while the LNCS group showed no weight gain. The LNCS group showed no compensatory increase in intake.
  In a 10-week trial conducted by Raben et al., (2002) [100], healthy males and females aged from 20 to 50 years and with a BMI of 25 to 30 kg/m^2^ were recruited. They were assigned to one of two groups: the first received foods and drinks containing LNCS (*n* = 20) and the other received equivalent products but sweetened with sucrose (*n* = 21). About 80% of the supplements were liquids. At the end of the trial, those consuming supplements with LNCS did not show changes in energy intake or macronutrient composition and did not gain weight. In contrast, the group with sucrose supplements, as it is to be expected, increased caloric intake by 10%, increasing weight and fat percentage significantly.
(b) *Mid- and long-term RCTs*. Theoretically, long-term studies represent the ideal scenario for studying complex phenomena such as weight change or the incidence of metabolic variables. Longer duration studies have been performed on “outpatient” subjects, without being confined to a controlled research facility. The disadvantage is that the researcher does not have complete control of the subject’s diet.
  Studies of more than 6 months duration for weight loss comparing foods or beverages sweetened with sugars contrasting them with their equivalents sweetened with LNCS replacing sugars generally show discreet but consistent and significant benefits in the reduction of weight for the LNCS equivalents [100]. In other trials, of the same or longer duration, using water as a comparator vs. drinks with LNCS, has yielded inconsistent results. That is, adding drinks “*on top of*” a plan for weight reduction, using either water or LNCS beverages (but without replacing sugars) some studies show a slight advantage in weight loss with water [101,102], and in others the advantage in weight reduction is reported in the group using beverages with LNCS [91]. It is possible that the effect of reduction in weight by adding liquids (either water or sweetened beverages with LNCS) depends more on the caloric restriction (of food in general) than on the effect of the non-caloric liquid accompanying or added to the diet. One of the longest studies identified was a 3-years old trial conducted by Blackburn et al. [103], which evaluated the use of LNCS in association with a program of weight loss and its maintenance. The sample consisted of 163 women with obesity (83 women completed the final phase of the study). At the end of the period of active maintenance (week 71), women belonging to the group with LNCS (aspartame) inclusion in the diet plan, showed a weight regain of 2.6 kg while the group that was allowed sugars had regained 5.4 kg. At the end of the second year of follow-up (week 175) subjects in the LNCS group regained 5.1 kg (keeping a 5% reduction with respect to their baseline weight loss) while the control group recovered all the weight lost initially, and ended up with their baseline weight.
(c) *Systematic reviews with meta-analyses*. Two recent meta-analyses, Miller and Pérez, 2014 [83] and Rogers et al., 2016 [84], analysed published clinical trials, reporting a minimal but favourable effect of LNCS reducing weight when used within a nutrition program replacing sugars. These systematic reviews and meta-analyses examined RCTs from very short term (1 day) to longer trials like those mentioned above, as well as observational studies. In Rogers et al., [84] all different types of studies were examined including animal and human intervention and observational studies, making this publication a very thorough review of the current literature. However, in 2017 Azad et al., published a new systematic review with meta-analysis of both RCTs and also prospective cohort studies [77]. This review only included studies of more than 6 months duration. This meta-analysis according to the authors “cannot support benefits of LNCS for the management of weight loss”. Inconsistent conclusions from available systematic reviews and their meta-analyses may result from including RCT’s developed to answer different research questions and/or experimental design.

Although controlled clinical trials are considered the “gold standard” design that evaluates cause-and-effect relationships and the effectiveness of any intervention or treatment in particular, they also exhibit clear limitations. Most have a small sample size with a lack of justification for its calculation. Others are built with cross-over design limitations, including the possibility of a residual effect between treatments; there is often no information about whether there a wash out period included. Another constraint is that many of published clinical trials are of short or very short duration [104,105,106,107,108,109,110,111,112].

### 4.4. Diabetes and Other Chronic Conditions

In the case of a possible association between diabetes (and other chronic diseases associated with cardiovascular risk factors) and the use of LNCS, there is a similar pattern in findings to those observed for investigations into weight loss, when only the observational studies are considered [110]. But when the best quality controlled clinical trials are evaluated [106,113,114,115,116,117,118,119], an overwhelming majority of them demonstrate a neutral effect of LNCS on relevant outcomes such as HbA1C, insulin and fasting or postprandial glucose. When LNCS are indicated within a nutritional plan structured in such a way as to replace sucrose, even discrete benefits can be seen at the levels of these parameters. It seems evident that observational and intervention studies report contradictory associations between LNCS consumption and metabolic outcomes [112]. The long-term epidemiological studies on the risk of developing type 2 diabetes show heterogeneous results [120], but most robust studies do not report any effects, even on lipid profile [121].

### 4.5. Key Points: LNCS Role in Body Weight Management and in Chronic Diseases

- The ad libitum consumption of LNCS, without being used to replace sugar/ reduce calorie intake from sugar, shows inconsistent results, i.e., seems to have no beneficial or detrimental effect on body weight.
- The use of LNCS in programs of weight reduction, replacing sucrose or simple sugars by LNCS may favour weight loss and weight maintenance. These “programs” should consider structured diet plans, (possibly monitored by healthcare professionals) as we well as an active, healthy lifestyle that includes a sensible, balanced diet and regular physical activity.
- LNCS-use in diabetic patient control programmes may contribute to better glycaemic control.
- Observational studies investigating the impact of LNCS on health outcomes should implement more robust intake assessments that facilitate the determination of overall LNCS intake, as well as intakes of individual LNCS. A potential approach may be to implement a biomarker approach [97].
- All results of observational studies must be considered in the light of evidence from intervention and safety research studies where biological outcomes can be measured directly.
- LNCS can provide dental health benefits, as studies show that products containing LNCS can reduce the risk of tooth decay [122,123].

## 5. Dietary Guidelines for LNCS

### 5.1. Background

Based on current scientific evidence and recent WHO recommendations [124], as well as guidance from other scientific bodies, this consensus statement recommends the reduction of free sugar intake to less than 10% of energy intake.

WHO suggests a further reduction of the intake of free sugars to below 5% of total energy intake (conditional recommendation). WHO defines “conditional recommendation” as those recommendations that are made when there is less certainty “about the balance between the benefits and harms or disadvantages of implementing a recommendation”. This means that “policy-making will require substantial debate and involvement of various stakeholders” for translating them into action. This recommendation aligns with the more recent recommendations of the UK Scientific Advisory Committee on Nutrition [125].

The use of LNCS in product reformulation could be a successful and sustainable strategy to achieve this important public health objective. However, it is also necessary to consider that sugars have many other functional roles in food production such as binding water, increasing boiling temperature and altering the texture of food products. Therefore, given the many functionalities of sugars in foods and beverages, it is not always possible to eliminate or replace sugars without affecting the quality and stability of particular foods [126].

Adequate intakes (AI) for water have been estimated by different bodies, including the IoM in the US and EFSA in Europe. AI for water in adults varies between 2 L and 3 L; for young children AI range falls between 0.9 L and 1.7 L, amounts to be supplied by foods (20–30%) and beverages (70–80%).

Food-based dietary guidelines recommend a reduction in free sugar intake by limiting sugary foods and drinks. Food-based dietary guidelines and their pictorial icons published in the last 10–15 years include recommendations about drinking water in different ways, such as Spain, Portugal, Venezuela, Costa Rica, Uruguay, Argentina, France, Belgium, The Netherlands, UK, Germany or Finland [127,128,129,130,131,132].

More recently, dietary recommendations from several countries refer to foods and beverages containing LNCS as possible alternatives to products sweetened with caloric sweeteners to promote the reduction of free sugar consumption with some advising on amounts of such beverages (Belgium, UK, Spain [127,130,131]), or recommending avoidance in sub-groups such as children aged under 6 years and pregnant women (Belgium) [131]. An issue that has generated some controversy is whether the consumption of foods and beverages containing LNCS during pregnancy and in young children is safe. The American Academy of Nutrition and Dietetics regards LNCS intake in pregnancy and young children as safe if maintained within acceptable daily intake levels [12]. In contrast, the US Institutes of Medicine (IoM) does not support the use of LNCS in children in the absence of evidence on long-term health effects, especially as a result of early childhood-onset exposure [15]. In the EU, the use of LNCS in infant formula, follow-up formula, baby food or/in dietetic foods intended for uses in young children is not permitted except where expressly indicated [133]. It has been suggested that consumption of LNCS in pregnancy could be associated with increased risk of preterm delivery, asthma, metabolic syndrome and diabetes in the offspring [134,135], although the evidence is also limited. According to regulatory authorities such as FDA and EFSA, the consumption of approved LNCS within the ADI is safe during pregnancy.

On a general basis, the intake of free sugars should be reduced as to account for less than 10% of total energy intake. Water is the beverage recommended to meet water intake needs, along with the water content of milk, fresh fruits and vegetables which should be consumed daily in adequate amounts as per food-based dietary guidelines.

Additional research, safety assessment and feasibility of new non- or low-calorie sweeteners from natural origin, either herbs, plants, fruits etc., and other possibilities offered by the immense flow of nature [136].

The consumption of foods and beverages with LNCS to replace those with added sugars can be an option to reduce sugar and energy intake, provided that the use of such products does not induce a compensation for energy intake and the general population should be advised on the role for such products in the context of a healthy diet and hydration.

### 5.2. Key Points: Dietary Guidelines for LNCS

- Dialogue with food and beverage manufacturers is required to discuss product reformulation to reduce the consumption of added sugars and/or replace total or partial content of sugar by LNCS.
- Given that sugars have many functional roles other than simply sweetening the product, reformulating with LNCS alone is unlikely to be appropriate for all types of products; therefore, other strategies to reduce free sugars intake such as reducing portion sizes should be considered in conjunction with reformulation.
- Many dietary guidelines and recommendations have been designed for adults only. It is recommended to prepare guidelines, with recommendations stratified for different age groups, with particular consideration for children and adolescents.
- Although there are no toxicological/safety concern or legal cut-off for children younger than 6 years, the consumption of foods and beverages with LNCS is unadvised within the context of education and sweet taste perception. LNCS however can be recommended in younger children with specific health conditions (diabetes, overweight, severe tooth decay history or any other condition where sugar intake restrictions is need).
- Foods and beverages containing LNCS can be recommended to replace foods and beverages with added sugars for patients with diabetes, dyslipidaemia, obesity or cardiovascular diseases.
- The use of LNCS is safe by pregnant women provided intakes do not exceed recommended intakes.
- Advice for lactating women is similar to that for pregnant women. Relevant local cultural aspects regarding recommendations for lactation and complementary feeding should be carefully considered.

## 6. Food and Nutrition Education and Consumer Behaviour

### 6.1. Background

Consumption of LNCS in foods and beverages has significantly increased in the last 30 years [137,138,139] and this trend is likely to continue with the implementation of strategies to meet public health goals in relation to reducing free sugars consumption. Strategies such as product reformulation to reduce the sugar content and energy density of foods and beverages will likely result in more widespread use of LNCS in the coming years [140].

Different terms are often used as synonyms for LNCS, which may have different meanings according to region, contributing to confusion among both professionals and consumers [141]. Though they are generally referred to as low-calorie sweeteners by the scientific community, they are sometimes also referred to as non-nutritive sweeteners, low energy sweeteners, intense sweeteners, high-potency sweeteners and sugar substitutes [142].

In recent years, the intake of LNCS has been investigated in the US, UK and in other countries using data from population studies. Several studies that have assessed the dietary intake of LNCS in European populations have consistently concluded that intake of LNCS is well below the ADI even for high consumers [56,58,59]. According to the results of Sylvetsky et al., [139], based on the information gathered in NHANES in the US, 25.1% of the children and 41.4% of adults reported LNCS use, in most cases (80% of children and 56% of adults) on a daily basis. In addition, most of the consumption events were in the home (71% in adults and 72% in children).

Hedrick et al. (2017) [143] considered intakes of LNCS from foods and beverages in adults in a rural area of Virginia (US) and found that the main dietary sources of LNCS were LNCS added at the table or in food preparation (37%) followed by diet tea (34%) and low-calorie soft drinks (27%). However, low-calorie soft drinks were the most frequently consumed dietary source of LNCS.

In Irish pre-schoolers aged 1 to 4 years, estimated intakes of four LNCS (acesulfame K, aspartame, saccharin and sucralose) did not reach risk levels, even among high consumers. One of the main sources of LNCS in this population group was flavoured drinks [57].

Also, in the US, Drewnowski and Rehm (2015) [144] reported higher consumption in the population with lower obesity burden and associated chronic diseases, and higher intake in individuals with higher educational and economic levels. These authors estimated that 30% of adults in the US consumed some type of LNCS, 19.5% from beverages, 11.4% added at the table and 4.6% from foods.

In the UK, based on information collected in the National Diet and Nutrition Survey, it was estimated that 44% of soft drinks consumed by adults aged between 19 and 64 years were low-calorie drinks (i.e., containing LNCS), which was identified to be a higher proportion than in other European countries [90].

Of particular note, a recently published study reported that even in subjects who reported not having consumed food or beverages with LNCS, sucralose was detected in urine, suggesting that other non-dietary sources, such as personal care products, e.g., such as toothpaste or medications, which may also contribute to overall exposure [145]. This study indicates that more comprehensive and reliable exposure assessments are required when investigating potential health impacts of LNCS consumption within free-living populations. It is important to clarify that when each regulatory body approves a LNCS that a proper exposure assessment should be performed, taking into account exposure to all products where the LNCS are used to ensure that the ADI is not exceeded, particularly by extreme users.

These points all lead to uncertainty in professionals and consumers, necessitating up-to-date and regular estimates of intakes in different population subgroups such as children (See Table 1) [146].

Interdisciplinary collaboration, especially among health professionals and communication professionals, is advisable, through associations of health informers and communication agencies. The interaction and two-way communication between health professionals and citizens are fundamental in the society of digital communication and new technologies. In this sense, the proper management of social networks through community managers capable of maintaining close contact with opinion leaders can be of great interest and impact. It is also very important to generate forums and dynamic channels of exchange of opinions, formulate concerns and generate answers that suit the changing needs and concerns of consumers. In this sense, the involvement of consumer associations, patient associations and other citizen organisations at different levels, with the involvement of local leaders, is essential.

### 6.2. Key Points: Food and Nutrition Education and Consumer Behaviour

- Consumers often find multiple sources of information on food and health issues, which do not always provide quality, transparent, sufficiently contrasted, and reliable information. In addition, the increasing use of new technologies and social networks favour the rapid diffusion and exchange of information, not all of which is reliable.
- It is necessary to facilitate access to information that is of proven quality, evidence-based, transparent, and easy-to-understand by the general public, to support warranted recommendations for their use.
- Sensory education, identification and recognition of flavours, taste, aroma and texture of foods and beverages plays a very important role in food education, which should be enhanced both in the family and in educational settings from early ages. In this context, recognizing the high preference for sweet taste, it would be desirable to educate the preference for lower sweetness intensity. This measure would be desirable both as an educational strategy during childhood, as in-patient’s education and dietary counselling.
- Continuing education of health professionals is required, since they are a source of reference information on issues related to food and health, for both the general population and patients. In this sense, the publication of positioning statements and consensus documents in academic reference publications may be adequate and convenient.
- It may also be appropriate to disseminate information through periodical bulletins, professional forums, scientific meetings, congresses and other regular communication channels established by scientific and professional societies in different health-related fields.
- Accredited reference websites can be interesting media, both to reach consumers and practitioners.

## 7. Conclusions and Key Messages

The experts of the Lisbon Consensus analysed and evaluated the evidence in relation to the role of LNCS in food safety, their regulation globally and the nutritional and dietary considerations of their use in foods and beverages. The main conclusions and key messages of each area were:

- **Safety of LNCS and monitoring systems**: LNCS are some of the most extensively evaluated substances in the human food chain. The safety of currently used LNCS has been reviewed and confirmed by health regulatory agencies globally, such as the FDA and EFSA.
- **Food composition and nutrition labelling for LNCS**: Consumer education about these products must be strengthened in a rigorous, objective way, based on the best scientific evidence and regulatory processes.
- **LNCS role in body weight management and chronic diseases**: The use of LNCS in programs of weight reduction replacing sucrose or simple sugars by LNCS may favour the reduction of excess weight and maintenance of weight loss in the context of structured diet plans. Furthermore, their use in diabetes control programme, replacing sucrose or simple sugars, may contribute to a better glycaemic control. These substances also provide dental health benefits.
- **Dietary guidelines for LNCS**: It is suggested that foods and beverages with LNCS are included as alternative options to sugar use in dietary guidelines.
- **Food and nutrition education and consumer behaviour**: Since health professionals are an important source of information for both the general population and patients on issues related to food and health, continuing education on the safety of and the regulatory processes around LNCS this group is necessary. In this sense, the publication of positioning statements and consensus documents in academic reference publications is highly desirable.

## Figures and Tables

**Table 1:** General characteristics of low and no-calorie sweeteners approved by the Food and Drug Administration (FDA) in the US and by the European Commission.

International Numbering System	Sweetener	ADI (FDA) (mg/kg Body Weight)	ADI (EFSA) (mg/kg Body Weight)	Sweetening Power ^1^	Discovery Date	Start of Use in EU	Chemical Structure
950	Acesulfame K	15	9	200	1967	1983	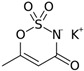
951	Aspartame	50	40	200	1969	1983	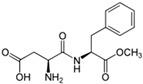
952	Cyclamates (Cyclamic acid and its Na and Ca salts)	Not approved	7	30–40	1937	1954	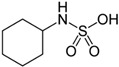
954	Saccharin and its Na, K and Ca salts	15	5	300–500	1879	1887	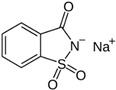
955	Sucralose	5	15	600–650 ^2^	1976	2000	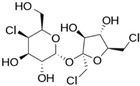
960	Steviol Glycosides	4 ^3^	4 ^3^	200–300	1931	2010 (Periodical JECFA evaluations since 2004)	RebaudiosideA 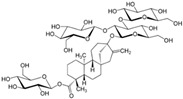
969	Advantame	32.8	5	37,000 ^4^		2013	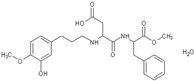
961	Neotame	0.3	2	7000–13,000 ^5^	1990	2010 2002 FDA	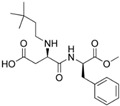
956	Alitame	Not approved	1	2000	1980	1996	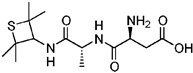
957	Thaumatin	Not approved	Not specified ^6^	2000–3000	1979	1999	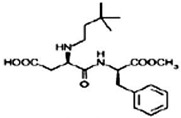
959	Neohesperidine Dihydrochalcone	Not approved	5	1.500	1960	1994	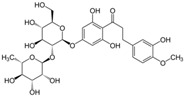

FDA: Food and Drug Administration; ADI: Acceptable Daily Intake; EFSA: European Food Safety Authority; ^1^ Sweetening power: modified from Carocho et al. (2017) [29], Difference of sweetness among different molecules, calculated based on the assumption that Sucrose is equivalent to 1 unit of sweetness. Data extracted from Mitchell (2006) [30]; Otabe et al. (2011) [31]; Varzakas et al. (2012) [32], European Union Regulation (EU) No 231/2012 (2012) [33]. ^2^ European Commission (EC) sucralose (2000) [34]. ^3^ Expressed in steviol equivalents. ^4^ European Union (EU). Regulation No. 497/2014 (2014) [4]. ^5^ EFSA (2007) [35]. ^6^ EFSA (2015) [36].
